# *Metaphycus macadamiae* (Hymenoptera: Encyrtidae) – a biological control agent of macadamia felted coccid *Acanthococcus ironsidei* (Hemiptera: Eriococcidae) in Hawaii

**DOI:** 10.1371/journal.pone.0230944

**Published:** 2020-04-08

**Authors:** Andrew Polaszek, John S. Noyes, Stephen Russell, Mohsen M. Ramadan

**Affiliations:** 1 Dept of Life Sciences, Natural History Museum, London, England, United Kingdom; 2 Core Research Laboratories, Natural History Museum, London, England, United Kingdom; 3 Division of Plant Industry, Plant Pest Control Branch, Hawaii Department of Agriculture, Honolulu, Hawaii; Instituto Federal de Educacao Ciencia e Tecnologia Goiano - Campus Urutai, BRAZIL

## Abstract

A new species of encyrtid wasp, *Metaphycus macadamiae* Polaszek & Noyes **sp. n.,** (Hymenoptera: Encyrtidae: Encyrtinae) is described as a solitary endoparasitoid of the invasive macadamia felted coccid, *Acanthococcus ironsidei* (Hemiptera: Eriococcidae) in Hawaii. This parasitoid is native to Australia, and the species description is based on material collected from a *Macadamia integrifolia* Maiden & Betche (Proteaceae) plantation in New South Wales, Australia, the native region of the host tree and insect. It is described here because it is a potential biological control agent against this pest where it has recently invaded Hawaii and South Africa.

## Introduction

Macadamia felted coccid (MFC), *Acanthococcus ironsidei* (Williams, 1973) (Hemiptera: Eriococcidae), is an Australian species, first found in Hawaii in 2005. On the Big Island, *A*. *ironsidei* has been found at Honomalino in South Kona. No infestations have been reported on the other neighbouring islands [1]. Host plants are restricted to smooth and rough-shelled macadamia *Macadamia integrefolia* and *M*. *tetraphylla*, respectively [2]. The species has become a problem in orchards where infested propagating material has been introduced, and where natural enemies do not keep it under control. Sometimes infested trees can be detected by a dull bronze colour in the foliage. In 2013 a species of the encyrtid parasitoid wasp genus *Metaphycus* was introduced from Australia into selected areas of Hawaii as an attempt at classical biological control of *E*. *ironsidei*. The new species is described below both to facilitate identification in the future, and to provide the formal nomenclature essential for all future work with this parasitoid.

Hawaii is the third largest producer of macadamia nuts in the world after Australia and South Africa. The macadamia nut industry is one of the top five agricultural commodities for the state. It is a vital part of the agricultural economy, with approximately 18,000 acres harvested on the Big Island of Hawaii, and farm value for the 2017–2018 crop is estimated at $53.9 million [3].

Host plants of *A*. *ironsidei* are restricted to macadamia [2, 4]. Adult females are immobile, resembling mealybugs, and lay their eggs within felted sacs that enclose their bodies. The life cycle takes approximately four weeks in Hawaii, and many overlapping generations are produced [5].

MFC is a severe pest of macadamia infesting all above-ground parts of trees, including the nut husks, and causing leaf malformation, discoloration and die-back of large parts of the tree [4, 6]. Heavy infestation causes death of young seedlings, reduction in nut production, and severe damage can eventually kill affected mature trees. Ironside [4] also mentioned that dense infestations could cause flower drop and subsequent reduction in nut setting.

MFC was initially found infesting macadamia trees at Honomalino, South Kona on the Big Island of Hawaii in February 2005. It is now expanding its distribution to Pahala and Paauilo throughout northern and eastern plantations. If not controlled, MFC will continue to threaten the entire macadamia nut industry in Hawaii. Recent state-wide surveys show that the other five Hawaiian islands are free of infestation.

In small to moderate sized trees, MFC infestations can be managed effectively using sprays of horticultural oils, a practice that has been used during outbreaks in Australia. Chemical control is expensive and potentially damaging to the environment, and most farmers in Hawaii would prefer not to spray. However, with the dense canopy in Hawaii’s orchards, the MFC populations appear to thrive, and local natural enemies are less common than in other areas. Imidacloprid root-drench application appears to be ineffective, and there are concerns relating to honeybee impact, as bees are commonly deployed for pollination in the orchards (Mark Wright, UH, personal communication).

Local predators and parasitoids may be helping to suppress the scale, but control at population level is not effective and needs to be enhanced by other selective parasitoids. Several extant natural enemies associated with MFC were observed in Hawaii including five species of predatory beetles, and the aphelinid parasitoid, *Encarsia lounsburyi* (Berlese & Paoli) [7]. Several entomopathogenic fungi kill *A*. *ironsidei* under laboratory conditions, but quantitative field studies are still pending [8].

Following a classical biological control approach, surveys in the native region to discover the key natural enemies suppressing MFC populations are essential for the Hawaii Department of Agriculture (HDOA) biocontrol program. MFC is less of a problem in Australia than in Hawaii, and specific natural enemies are thought to be an important mortality factor. The Plant Pest Control Branch (HDOA) considered that classical biological control could offer a long-term solution for suppression of MFC. In December 2013, HDOA initiated a foreign exploration to Australia to search for natural enemies of MFC. Macadamia and MFC are native to Australia, and therefore it was the most likely place for locating host-specific parasitoids. An encyrtid wasp, *Metaphycus* sp., was collected and shipped for host specificity tests in the HDOA Insect Containment Facility. Morphology-based identification (by JSN) revealed the species to be undescribed, and this was later confirmed by sequencing of two gene fragments, partial mitochondrial CO1 and ribosomal 28sD2. The new species is described below both to facilitate identification in the future and to provide the formal nomenclature essential for all future work with this parasitoid.

## Materials and methods

### Specimen depositories: Abbreviations

ANIC: Australian National Insect Collection, CSIRO, Canberra, Australia.

BPBM: Bernice P. Bishop Museum, Honolulu, Hawaii.

NHMUK: Natural History Museum,. London, UK.

USNM: United States national Museum, Washington D.C., USA.

### Collection

In November 2013, a survey was undertaken by MMR in Alstonville, NSW, Australia Australia (28°51’ 20.14”S, 153°26’31.40”E), where *Metaphycus* was dissected from MFC infested leaves of *Macadamia integrifolia*. Two shipments of infested macadamia leaves were collected and shipped to Hawaii. Infested leaves were taken from different trees that were not known to be sprayed, and these leaves produced adult *Metaphycus* wasps. A colony was initiated from 55 founder *Metaphycus* adults reared on seedlings infested with MFC at the HDOA Insect Containment Facility (Honolulu). The colony is still active, and wasps are currently used to conduct studies on host range and biology.

### Morphological study

Morphological terminology and the format for the species description follow Noyes [9].

Abbreviations are as follows: AOD = largest diameter of anterior ocellus; AOL = minimum distance between posterior ocellus and anterior ocellus; EL = eye length; EW = eye width; FV = minimum width of frontovertex; FVL = length of frontovertex from occipital margin to top of antennal scrobes as seen in dorsal view; FVS = width of frontovertex a little above top of scrobes at a point where eye margin changes from being virtually straight to distinctly curved; FWL = fore wing length; FWW = fore wing width; GL = gonostylus length; HW = head width measured in facial view; HWL = hind wing length; HWW = hind wing width; MS = malar space (minimum distance between eye and mouth margin); MT = mid tibia length OCL = minimum distance between posterior ocellus and occipital margin; OL = ovipositor length; OOL = minimum distance between eye margin and adjacent posterior ocellus; POD = largest diameter of posterior ocellus; POL = minimum distance between posterior ocelli; SL = scape length; SW = scape width.

Card-mounted specimens were observed with a Leitz Dialux binocular microscope at magnifications ranging from 20-80x. Slide-mounted specimens were observed with a Leitz Dialux 20 microscope at magnifications ranging from 40-400x.

**Fig 1:**
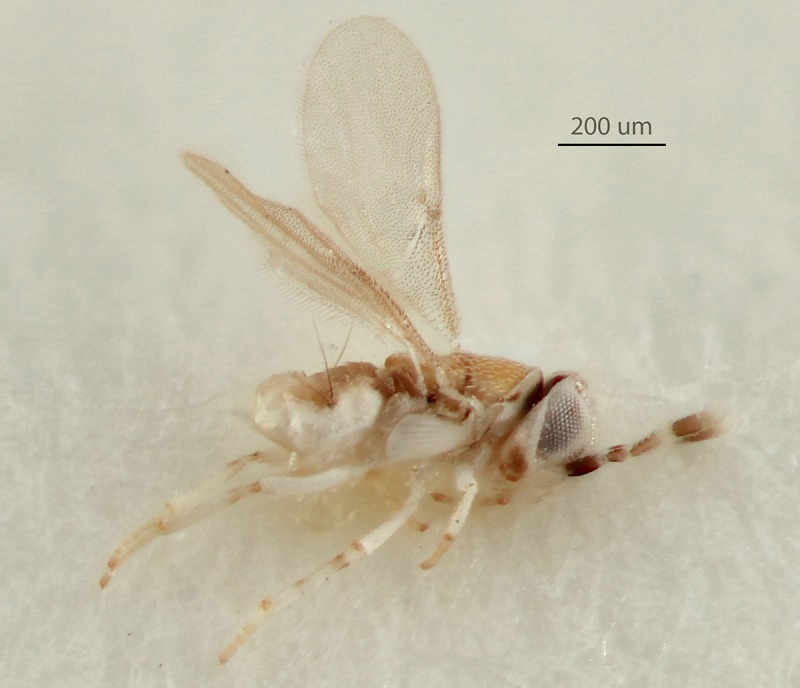
*Metaphycus macadamiae* female holotype, habitus (photo by N. Dale-Skey–specimen subsequently slide-mounted).

**Fig 2:**
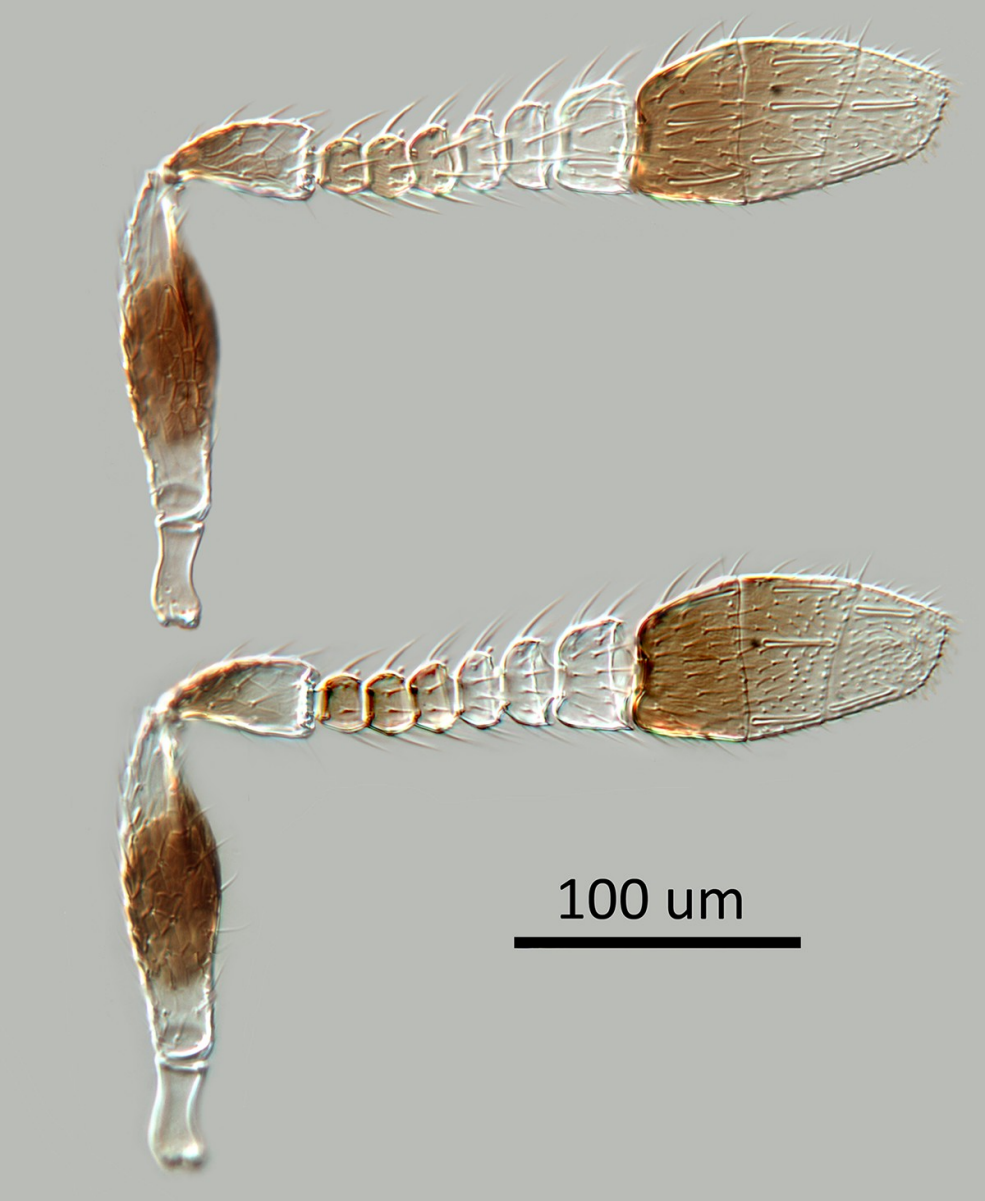
*M*. *macadamiae* female holotype, antenna, inner and outer aspects.

**Fig 3:**
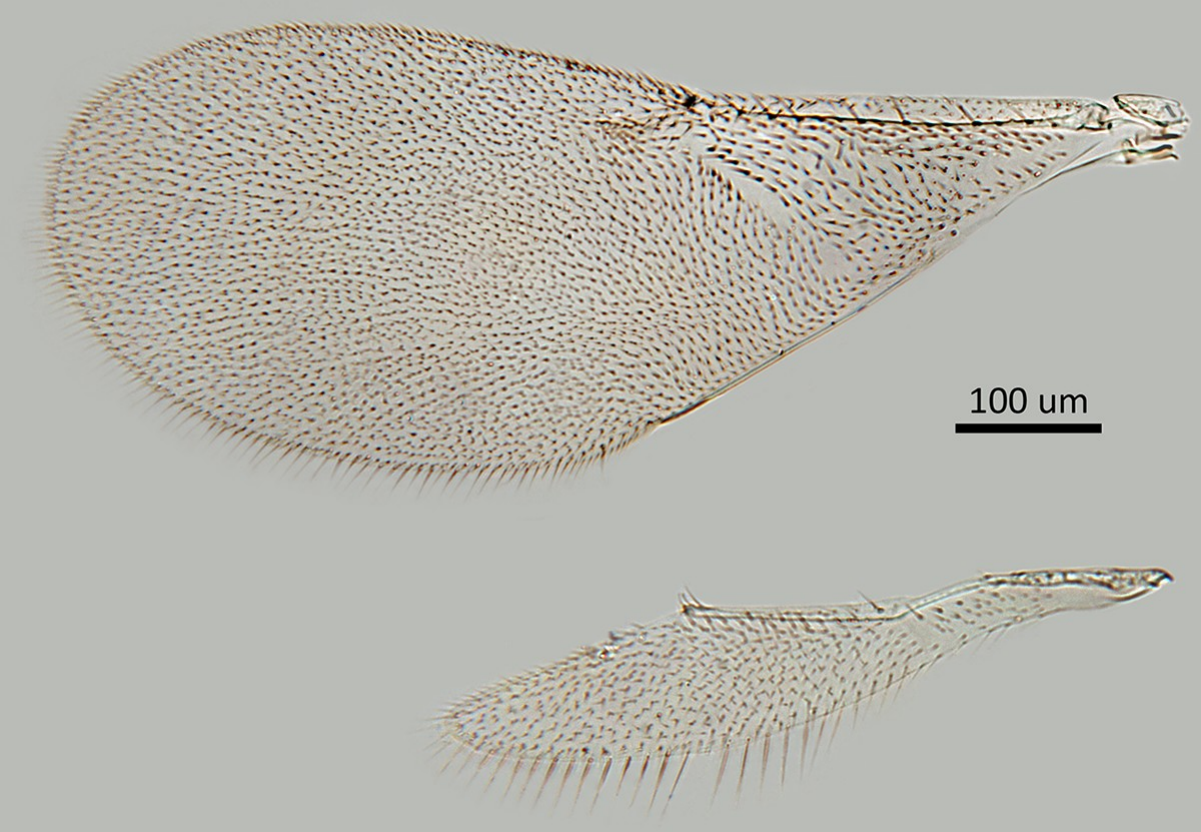
*M*. *macadamiae* female holotype, fore and hind wings.

**Fig 4:**
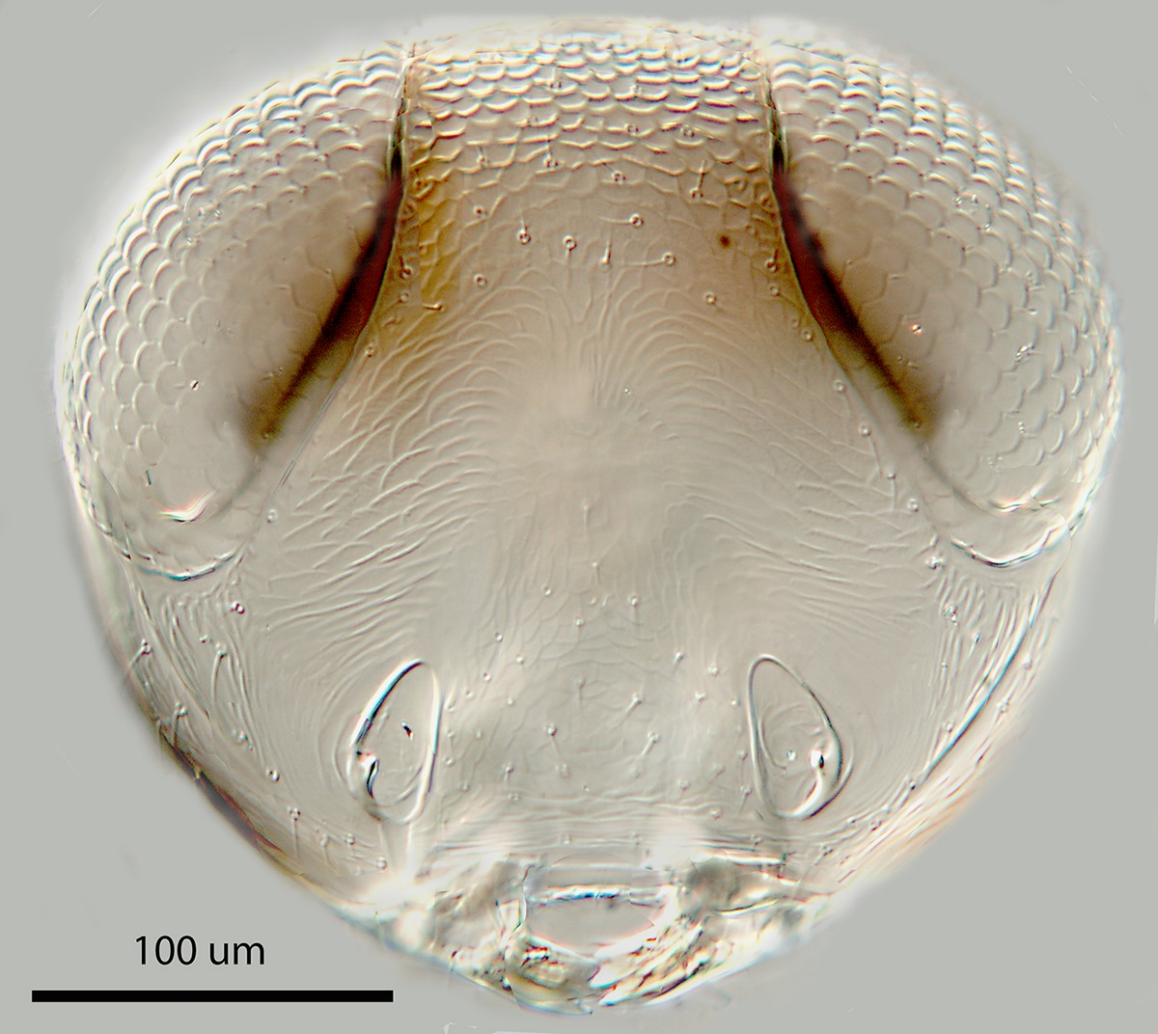
*M*. *macadamiae* female holotype, head, frontal view.

**Fig 5:**
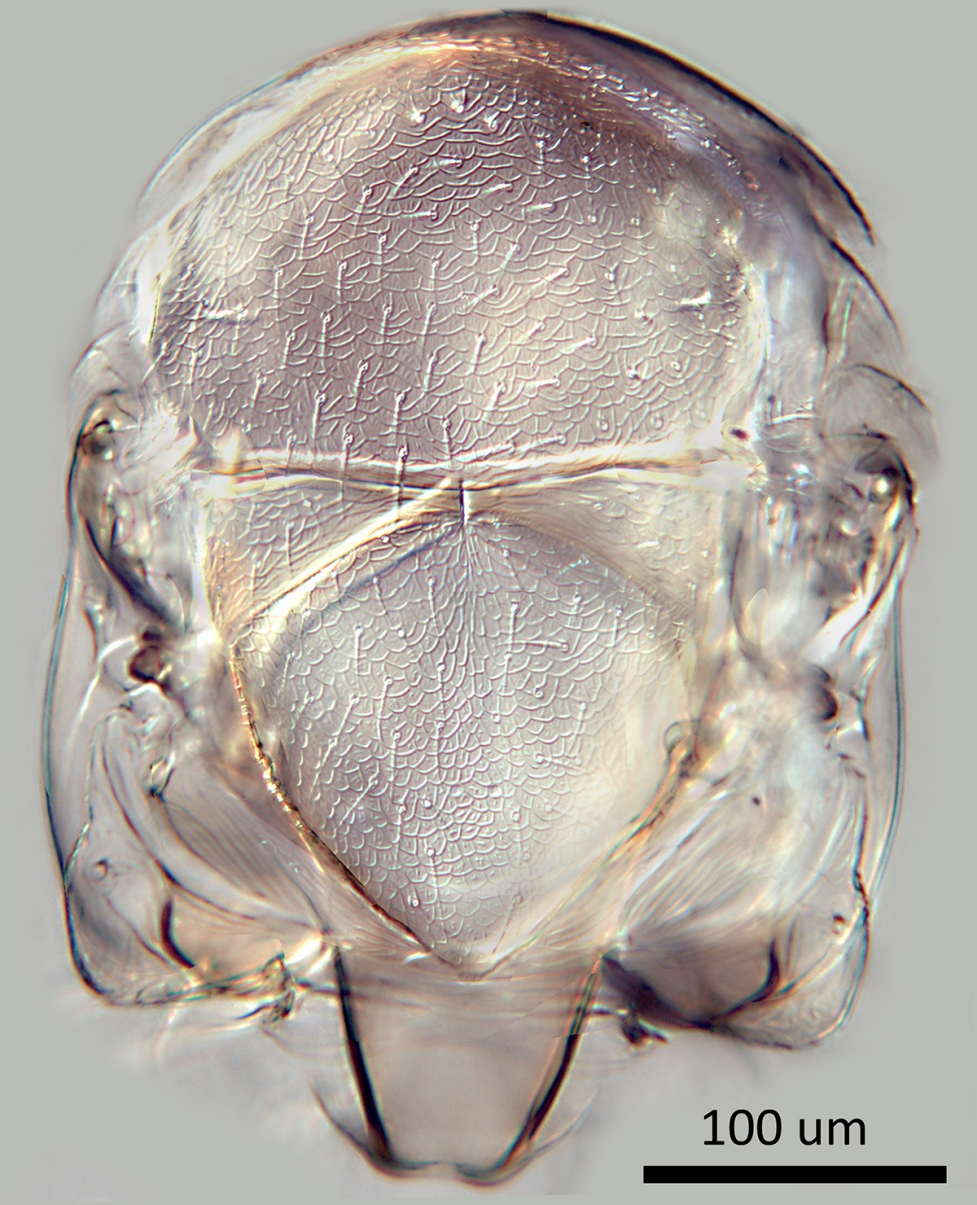
*M*. *macadamiae* female holotype, dorsal mesosoma.

**Fig 6:**
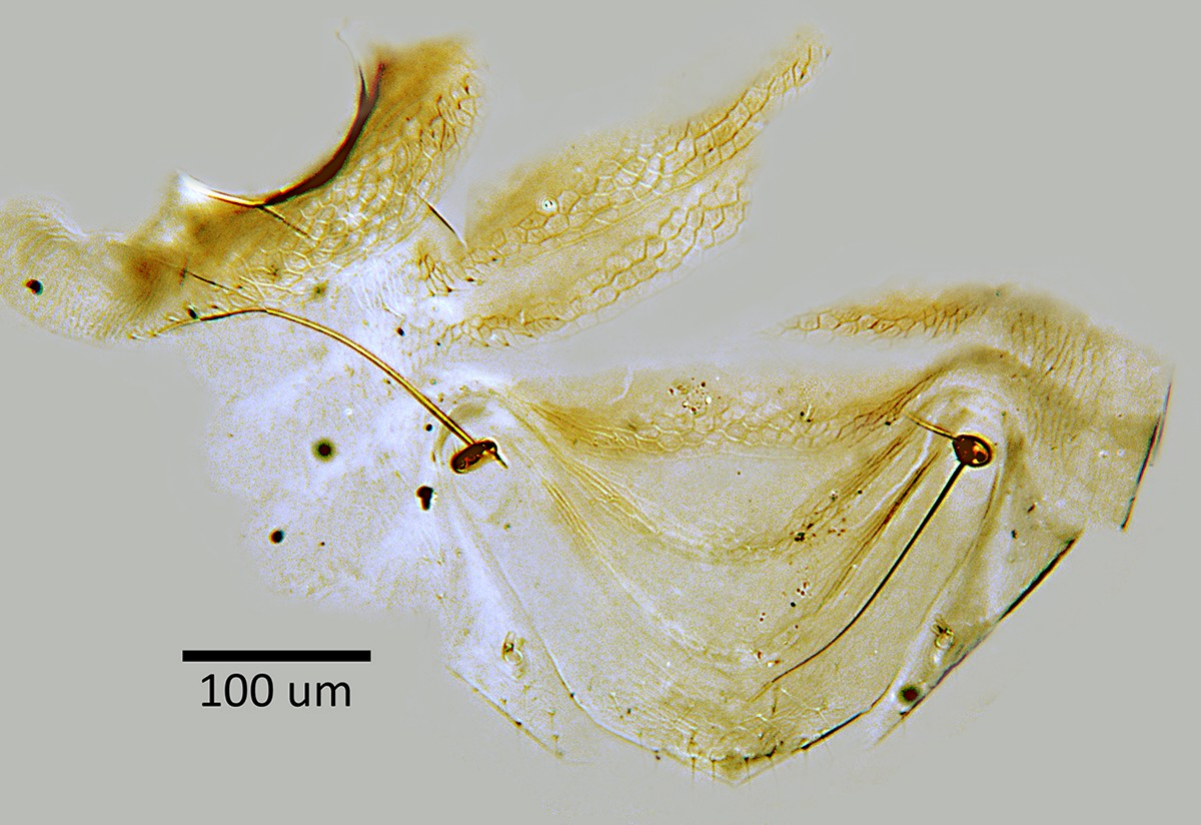
*M*. *macadamiae* female holotype, metasomal terga.

**Fig 7:**
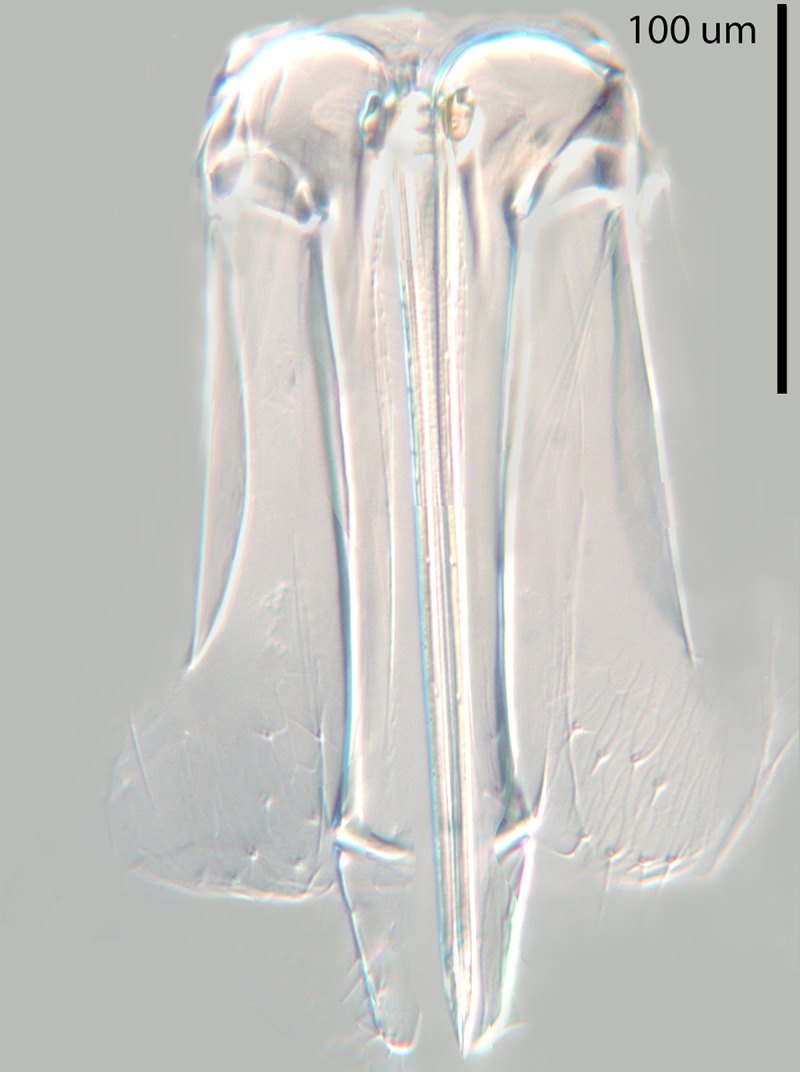
*M*. *macadamiae* female holotype, ovipositor.

**Fig 8:**
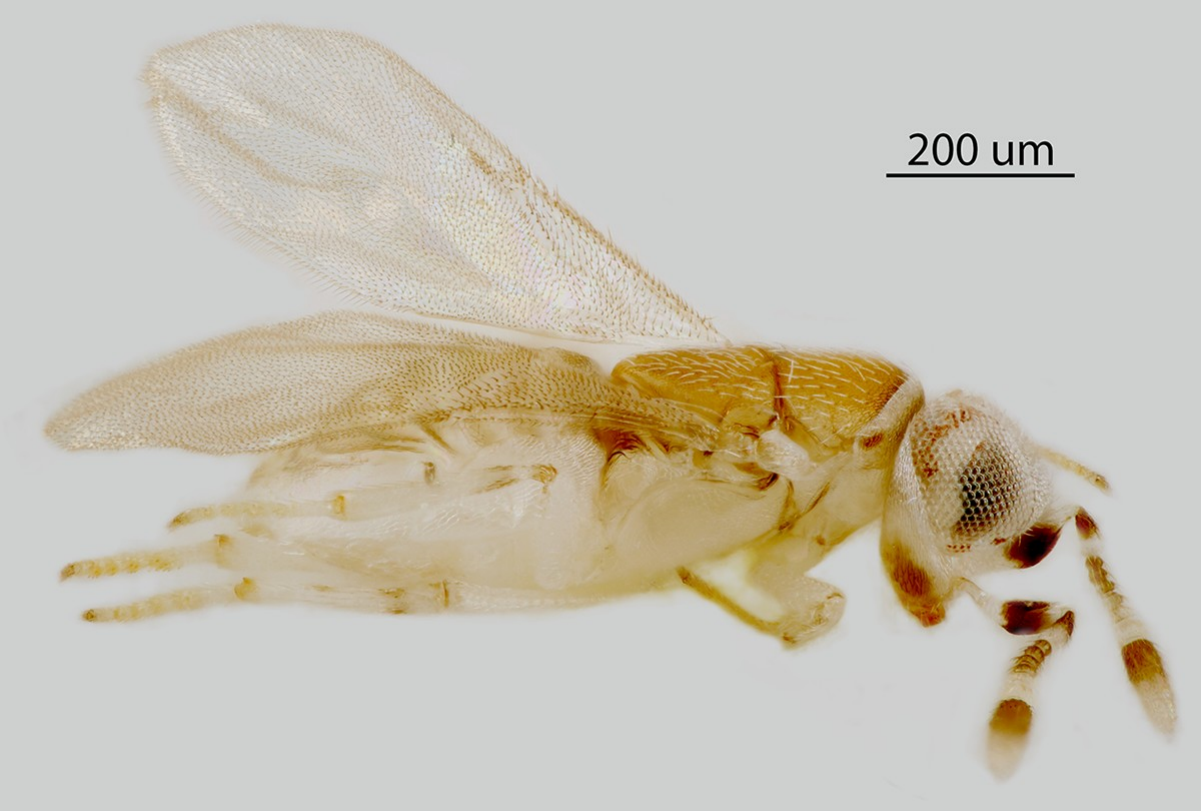
*M*. *macadamiae* female, lateral habitus.

**Fig 9:**
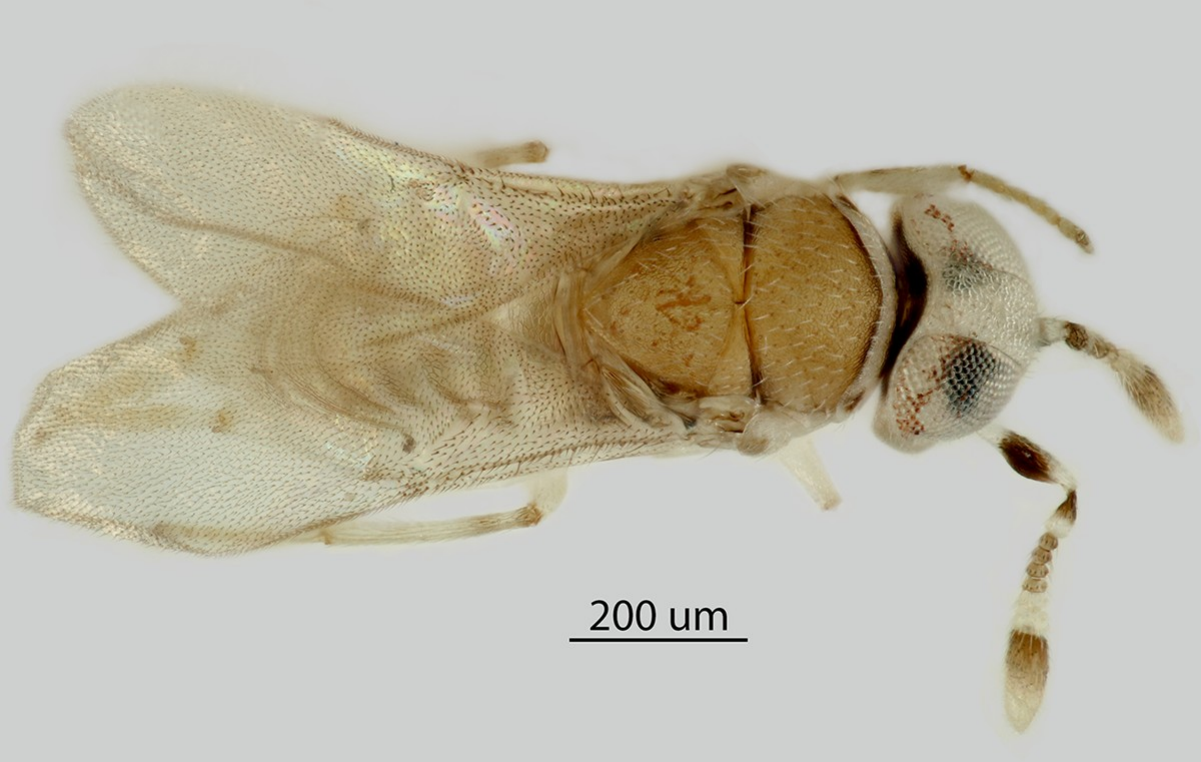
*M*. *macadamiae* female, dorsal habitus.

**Fig 10:**
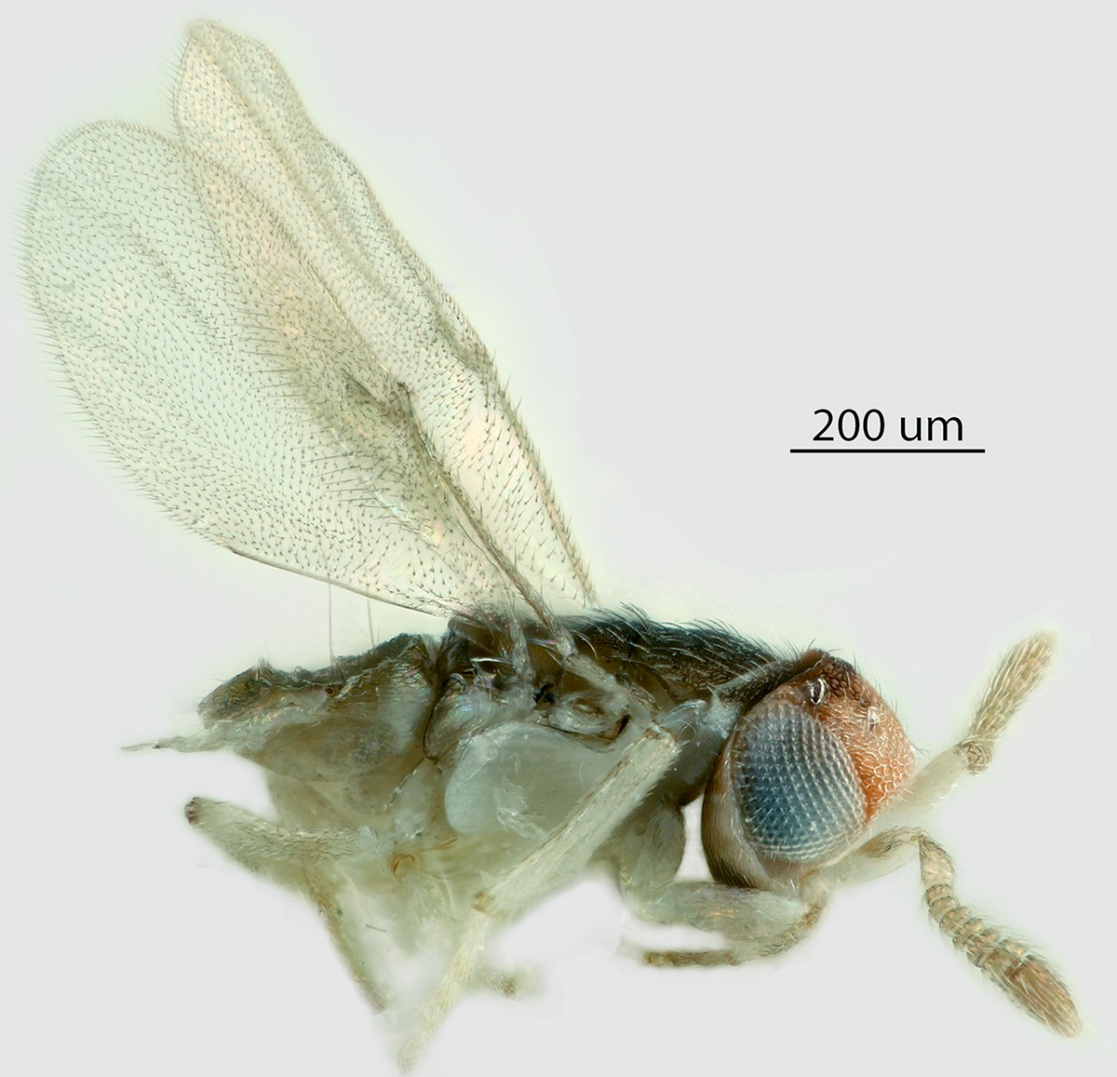
*M*. *macadamiae* male, lateral habitus.

**Fig 11:**
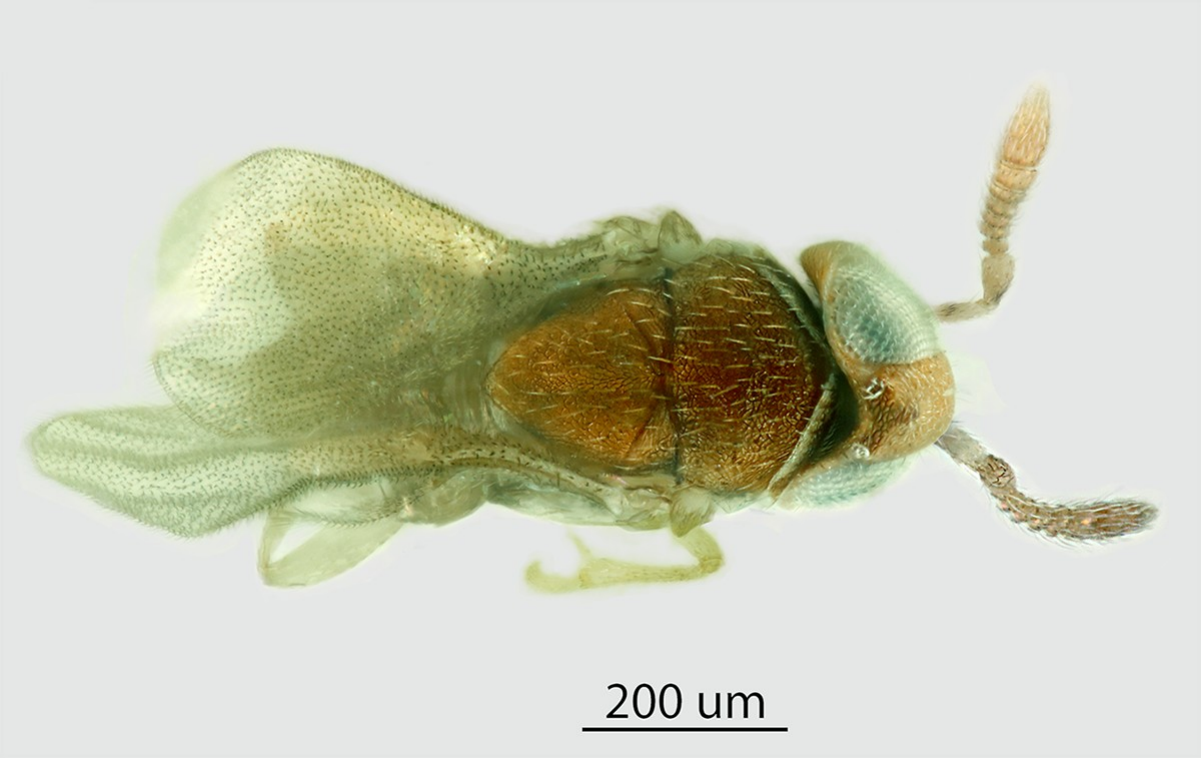
*M*. *macadamiae* male, dorsal habitus.

**Fig 12:**
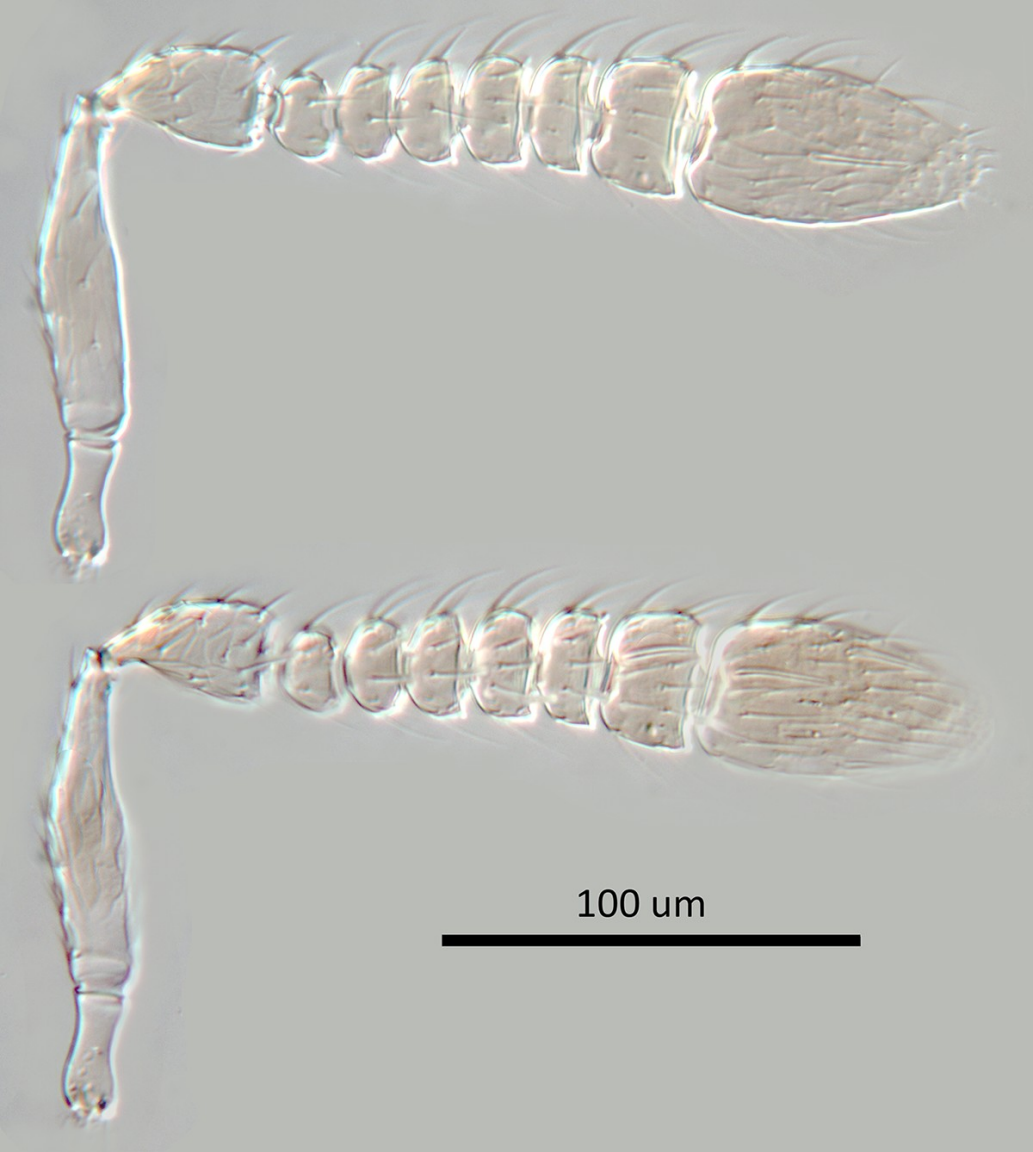
*M*. *macadamiae* male antenna, inner and outer aspects.

**Fig 13:**
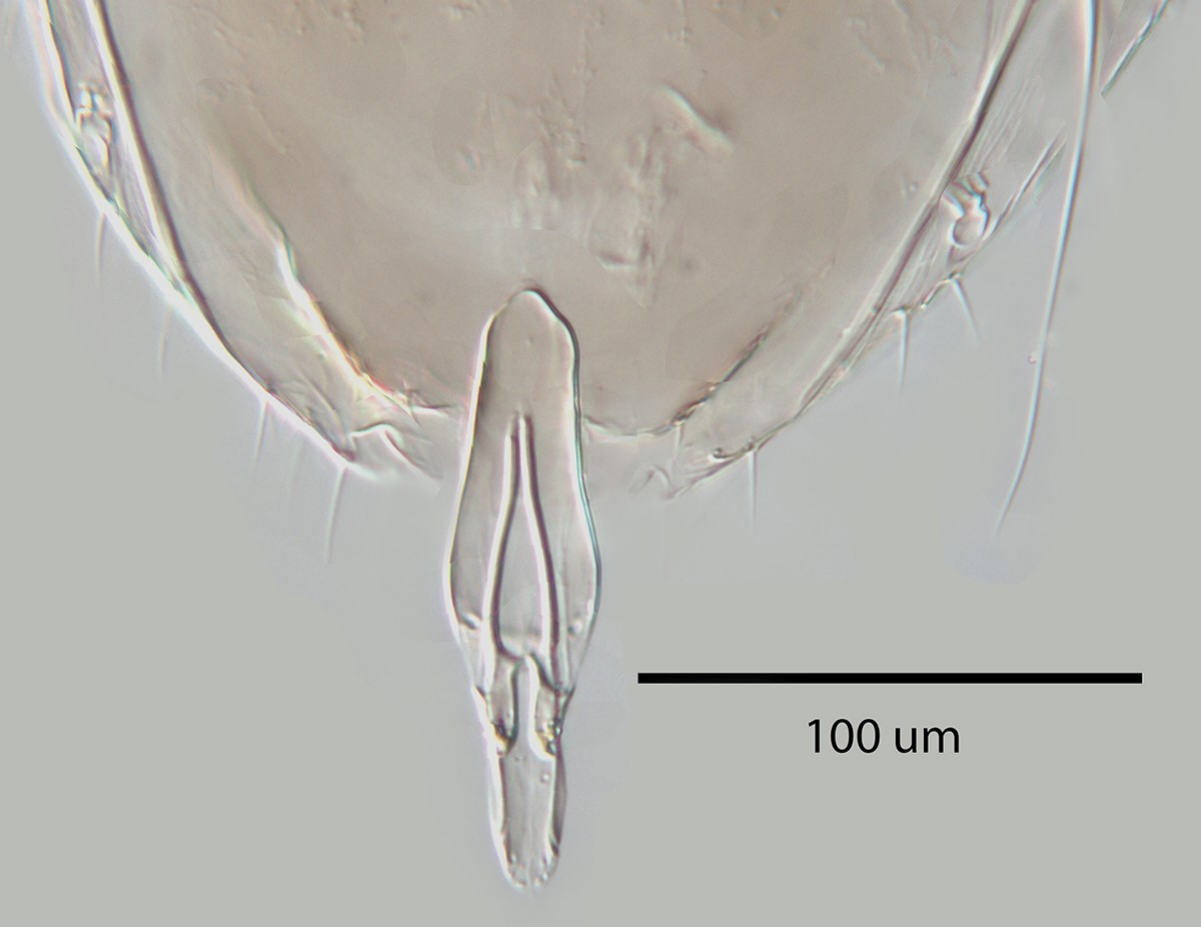
*M*. *macadamiae* male genitalia.

Images were generated as follows: Fig 1 (Holotype habitus: Canon DSLR with 100 mm macrolens, processed with HeliconFocus stacking software with final editing in Adobe Photoshop CC. Figs 2 & 3: Canon DSLR with 10x Mitutoyo objective, processed with HeliconFocus stacking software with final editing in Adobe Photoshop CC. Figs 4–13 Leitz Dialux 20EB compound microscope using Nomarski Differential Interference Contrast illumination, photographed with MicroPublisher 5.0 RTV camera; scanned sections stacked and combined using Synoptics AutoMontage^®^ software, and final images edited with Adobe Photoshop CC^®^.

### DNA sequencing

Genomic DNA extraction was undertaken using the protocol in Polaszek *et al* [10] and Cruaud *et* al. [11], which leaves the sclerotized parts of the specimen intact. Specimens were then critical point dried and card-mounted, with selected individuals then dissected and mounted in Canada balsam on microscope slides.

As the Folmer primer pair LCO1490/ HCO2198 [12] does not perform well in many chalcid wasp taxa [13–15], especially in those with suboptimal DNA extracts [16], a shorter than standard CO1 sequence was obtained of 555 bp after trimming the primer sequences and poor-read ends. The 28S D2 fragment was amplified with the primers D23F (5′-GAG AGT TCA AGA GTA CGT G-3′) [17] and 28Sb (5′-TCG GAA GGA ACC AGC TAC TA-3′) [18, 19]. After trimming the primer sequences and poor-read ends, the resulting contig from 7 forward and 8 reverse sequences was 444 bp. All reactions were carried out in 25 μl reaction volume containing 5 μl of template DNA, 2.5 μl of 10× PCR buffer, 0.75 μl of 50 mM MgCl2, 0.2 μl dNTPs solution (25 mM each), 1.25 μl of each primer (10 μM), 0.3 μl Taq polymerase (5u/μl Biotaq, Bioline), and PCR grade water to final volume. The PCR cycle for the 5’ end of the standard barcode region consisted of an initial denaturation step of 94°C for 2 min, followed by 40 cycles of 94°C for 30 s, 40°C for 60 s and 72°C for 30 s, and a final extension step of 10 min at 72°C. For the 3’ end of COI and for 28S the conditions where similar except for annealing at 41°C for 50 s and 55°C for 30 s respectively.

Both DNA strands were sequenced at the Natural History Museum Life Sciences DNA Sequencing Facility (London) using the same primers used for the PCR. Forward and reverse sequences were assembled and corrected using Sequencher version 4.8. Identical partial sequences were obtained for 8 individuals for 28S, and 3 individuals for CO1. These have been deposited in Genbank under accession nos MN933670 (CO1) and MN934351 (28S), respectively.

### Nomenclatural acts

The electronic edition of this article conforms to the requirements of the amended International Code of Zoological Nomenclature, and hence the new names contained herein are available under that Code from the electronic edition of this article. This published work and the nomenclatural acts it contains have been registered in ZooBank, the online registration system for the ICZN. The ZooBank LSIDs (Life Science Identifiers) can be resolved and the associated information viewed through any standard web browser by appending the LSID to the prefix "http://zoobank.org/". The LSID for this publication is:

urn:lsid:zoobank.org:pub:E842825D-2E5A-47C4-AD7B-2A742D3347C8

The electronic edition of this work was published in a journal with an ISSN, and has been archived and is available from the following digital repositories: PubMed Central, LOCKSS.

### Description

#### Metaphycus Mercet

Metaphycus Mercet, 1917:138. Type species: Aphycus zebratus Mercet, by monotypy, as subgenus of Aphycus Mayr.

*Metaphycus* Mercet, 1925:28. Generic status.

Synonyms include *Aenasioidea* Girault, *Aenigmaphycus* Sharkov & Voynovich, *Anaphycus* Sugonjaev, *Erythraphycus* Compere, *Euaphycus* Mercet, *Mercetiella* Dozier, *Melanaphycus* Compere, *Mesaphycus* Sugonjaev, *Notoencyrtus* De Santis, *Ooaphycus* Girault, *Tyndarichoides* Girault and *Xenaphycus* Trjapitzin [20].

#### Diagnosis

Length 0.5–1.8 mm; robust and squat species, rarely slender and elongate; body largely orange, yellow to brown or black (they may be shiny), never with metallic lustre, antenna usually with black and white or yellow parts or segments, fore wing hyaline to partially or uniformly infuscate, legs yellowish or with brown to black segments, tibiae frequently with dark rings. Head with occipital margin sharp, frequently with shallow grooves lateral to outer margin of torulus; mandible mostly broad with 3 short, subequal teeth, but occasionally slender with two or three unequal teeth. Pronotum short, broadly triangular in dorsal view, mesoscutum wider than long, notaular lines variable in length from virtually absent to complete and reaching posterior margin; scutellum never with an apical flange that overhangs the propodeum medially; fore wing generally about 2.5X as long as broad and with uniform setation, submarginal vein reaching about half way along wing, marginal and postmarginal veins very short, stigmal vein well developed, longer than marginal and postmarginal veins together; linea calva interrupted in posterior third by a few setae, or completely closed at this point; mid tibial spur about as long as mid basitarsus, rarely significantly shorter. *Female*: antenna almost always 11-segmented (1163), rarely with clava 2- segmented; scape cylindrical to strongly expanded and flattened. Gaster with hypopygium reaching half way along gaster to more or less reaching its apex; outer plates of ovipositor not reflected upwards posteriorly; gonostylus free, in most cases not exserted or only slightly so. *Male*: generally darker and with more uniform colour in respect to that of corresponding female. Antenna 9-segmented (1161), with setae longer than in female; toruli very often with associated pores.

#### Comments

Females of *Metaphycus* that have the ovipositor slightly exserted may be confused with *Aphycus* (Mayr). In *Aphycus*, the linea calva of the fore wing is always clearly entire and the outer plates of the ovipositor are reflected upward posteriorly to connect loosely with the syntergum.

#### Distribution

Of the 466 described species of *Metaphycus* [21] three species are more or less cosmopolitan *(helvolus*, *lounsburyi* and *flavus)*, 80 are Afrotropical, 208 are Neotropical, 48 are Nearctic, 87 are Palaearctic (including 53 from Europe), 26 are Oriental, and 23 are Australasian, with several species being found in more than one region.

#### Hosts

*Metaphycus* species are mainly reported as solitary or gregarious parasitoids of soft scales (Hemiptera: Coccidae) (e.g. *Coccus*, *Ceroplastes*, *Saissetia* spp.) and diaspidids (Hemiptera: Diaspididae). A few species have been reported as parasitoids of kermesids (Hemiptera: Kermococcidae), asterolecaniids (Hemiptera: Asterolecaniidae), kerrids (Hemiptera: Kerridae), eriococcids (Hemiptera: Eriococcidae), cerococcids (Hemiptera: Cerococcidae), mealybugs (Hemiptera: Pseudococcidae), whiteflies (Hemiptera: Aleyrodidae) and triozids (Hemiptera: Triozidae) [21].

#### Biocontrol

Species of the genus play an important role in the natural regulation of scale insect pests, and as a result nearly 30 species have been released in various parts of the world for control of soft scale (Hemiptera: Coccidae) and armoured scale (Hemiptera: Diaspididae) pests of agriculture. The use of *Metaphycus* species in biocontrol programmes has been summarized [20, 22], and a detailed compilation of data is available [21]. In general, the most successful introductions have been from southern Africa into California for the control of soft scale pests on *Citrus* with the best known of these being the release of *M*. *helvolus* (Compere) in 1937 for the control of *Saissetia oleae* (Olivier, 1791) (Hemiptera: Coccidae). This has been estimated to have saved the California citrus industry at least $70m prior to 1979, with an annual saving of over $2m [23]. The same species has proved to control successfully a number of *Saissetia* spp. virtually everywhere it has been released for pest control throughout the world.

#### Identification

Several keys have been published to the species of *Metaphycus*: African species [24], South African species [25–27], Central Asian species [28], Italian species [29], European species [20], Palaearctic species [30], Indian species [31], all of them based on the distinction of species groups using the palp formula [32]. Some of these keys are based largely on characters which may be unreliable (e.g. colour of funicle segments, very small differences in relative width of scape) or difficult to evaluate (e.g. relative length of frontovertex). Other character states that may prove useful in the identification of species are the presence or absence of subapical setae on the 2^nd^ valvifer, the presence or absence of lateral antennal grooves, the shape of the antennal scrobes and the structure of the ovipositor and shape of the hypopygium. Unfortunately, most of these characters can be observed only on well prepared slide-mounted material which makes the reliable identification of a number of species very difficult.

#### *Metaphycus* macadamiae Polaszek & Noyes sp. N

urn:lsid:zoobank.org:act:0668979C-A7BF-4600-B54C-82E1B5941743

**Figs 1**–**13**

#### Morphology

Female (holotype, Fig 1): length, including ovipositor, 0.76mm, excluding ovipositor, 0.73mm (critical point dried specimen).

Colour: Head mostly white with occiput above foramen dark brown and gena with a slightly elongate pale brown mark between base of mandible and eye; mouth margin with a slender brown mark above base of mandible and another below torulus; antenna (Fig 2) with radicle white; scape white with a broad median band about half its length on both inner and outer surfaces, connected ventrally but narrowly separated dorsally; pedicel brown in proximal half, distal half white; funicle with F1-F2 pale brown, F3 slightly paler, F4-F6 white; clava pale brown in proximal half, apex pale yellow; pronotum white with paired sublateral brown spots on posterior margin and a pair of larger submedian subtriangular marks on neck; mesoscutum (Figs 5, 8 and 9) pale orange with posterior margin narrowly dark brown adjacent to axilla; axilla and scutellum pale orange; metanotum dusky pale orange; tegula translucent white, apex pale grey; side and venter of thorax white; mesoscutum and scutellum clothed in numerous, moderately long, translucent setae; coxae and legs white to very pale yellow, mid and hind tibiae each with an extremely faint pale brown subbasal ring; fore wing hyaline, venation pale yellow; propodeum medially pale orange but pale orange-brown in lateral third towards spiracle, side white; dorsum of gaster slightly dusky pale orange with syntergum white, side and venter white; gonostylus white.

Morphology: Head 3.5x as wide as fronovertex, slightly shiny on frontovertex, sculpture coarse and fairly regularly reticulate, of mesh size hardly smaller than eye facet; ocelli forming an angle of about 43°; antenna (Fig 2) with scape about 2.9X as long as broad; F1-F5 subequal and transverse but increasing very slightly in width distad, F6 clearly longer and larger; funicle with linear sensilla only on F6; clava apically rounded; eye slightly overreaching occipital margin; upper temple rounded in facial view; frontovertex hardly less than one-third head width, with inner eye margins diverging slightly anteriorly, with narrowest point about level with posterior ocelli; scrobes deep, U-shaped, meeting dorsally, interantennal prominence dorsally rounded; lateral antennal groove absent; antennal torulus separated from mouth margin by slightly less than its own length; mandible broad with three short, more or less equal, acute teeth; palp formula 2–2. Relative measurements: HW 64, FV 21, FVS 20, FVL 35, POL 7, AOL 10, OOL 1.5, OCL 2, POD 4, AOD 4, EL 36, EW 29, MS 20, SL 28, SW 9.5.

Thorax with notaular lines absent externally, but visible anterolaterally on slide-mount; dorsum of thorax shiny with sculpture on mesoscutum similar to that of frontovertex, but shallower and composed of smaller cells, sculpture of scutellum about as deep as that on mesoscutum; side of propodeum more or less naked; fore wing venation and setation as in Fig 3. Relative measurements: FWL 165, FWW 66; HWL 110, HWW 21.

Gaster with ovipositor slightly exserted, the exserted part about 0.15X as long as gaster or 0.7X as long as mid tibial spur; gonostyli together cylindrical and proximally about 2X as deep as diameter of base of mid tibial spur; apex of last tergite shallowly rounded; hypopygium reaching about 0.6X along gaster, broadly subtriangular and about 2X as broad as long; second valvifer with 1 or 2 subapical setae. Relative measurements: OL 64, GL 13 [MT 58].

Variation. The overall length of the female varies from about 0.63–0.78mm and the head varies from about 3.0–3.6X as wide as the frontovertex.

Male (Figs 10–13): length 0.46–0.66mm. Structurally very similar to female except structure of antenna and genitalia.

Colour: Head mostly pale orange with occiput above foramen dark brown; frontovertex with a triangular brown mark delimited by occipital margin and anterior ocellus; gena and temple pale pink with posterior margin brown from base of mandible to about level of lower eye margin; mouth margin very narrowly margined brown; scrobal area very pale yellow; antenna (Fig 12) with radicle white; scape very pale yellow, almost white; pedicel brown in proximal half, distal half off-white; flagellum pale brown; pronotum very pale yellow with paired sublateral brown spots on posterior margin and a pair of larger submedian subtriangular marks on neck; mesoscutum, axilla and scutellum orange-brown; metanotum dusky orange; tegula white with apex brown; side and venter of thorax white; mesoscutum and scutellum clothed in numerous, moderately long, translucent pale brown setae; coxae and legs white to very pale yellow, mid and hind tibiae each with an extremely faint pale brown subbasal ring; fore wing hyaline, venation pale yellow; propodeum medially pale orange but pale orange-brown in lateral third towards spiracle, side white; dorsum of gaster orange-brown with syntergum slightly dusky pale orange, side and venter white.

Morphology: Head about 2.4–2.6X as wide as frontovertex with inner eye margins diverging slightly anteriorly; antennal torulus with from 1 to 4, widely spaced, associated pores along inner margin; antenna as in Fig 12 with scape about 2.7X as long as broad, F1-F5 anneliform, subequal, F6 largest and slightly transverse, only F6 with linear sensilla. Phallobase (Fig 13) about as long as aedeagus with a single subapical, seta on each side and each digitus with a single apical hook; aedeagus about 0.5X as long as mid tibia. Relative measurements (slide-mounted specimen): HW 61, FV 24, SL 22, MT 49.5, AL 23.5.

#### Hosts

A parasitoid of *Acanthococcus ironsidei* (Williams) (Hemiptera: Eriococcidae) on *Macadamia integrifolia* Maiden & Betche (Proteaceae).

#### Distribution

Australia (New South Wales), Hawaii (introduced).

#### Material examined

Holotype ♀, HAWAIIAN ISLANDS, Oahu, Pawaa, Hawaiian Dept. Agric. Insect Containment Facility, May 14 2015, lab reared *Eriococcus ironsidei* F18 generation (J. Yalemar), original collection AUSTRALIA, NSW, Alstonville, ex *Eriococcus ironsidei* on *Macadamia integrifolia* Tax. coll. #15–228; 19.xi.2013/26.xi.2013 and Tax. coll. #15–229 25.xi.2013/7.xii.2013 (M. Ramadan). Paratypes: HAWAIIAN ISLANDS, 9♀, 9♂, same data as holotype. Holotype in ANIC, paratypes in BMNH, BPBM and USNM.

#### Comments

The female of *Metaphycus macadamiae* has a unique combination of diagnostic characters in the genus: 2–2 palp formula; body generally white to pale orange with occiput and pronotum marked dark brown; clava proximally dark brown with apex pale yellow; head mostly white with occiput above foramen dark brown and gena with a slightly elongate pale brown mark between base of mandible and eye; scape white with a broad median band about half its length on both inner and outer surfaces, connected ventrally but narrowly separated dorsally; fore wing hyaline; legs white to very pale yellow, mid and hind tibiae each with an extremely faint pale brown subbasal ring; metanotum dusky pale orange; scape about 2.9X as long as broad; funicle with linear sensilla only on F6; head 3.0–3.6X as wide as the frontovertex; ovipositor about 5X as long as gonostylus.

Of the 30 or so species of *Metaphycus* that have been reared from Eriococcidae worldwide, only two belong to the *alberti* species group (both maxillary and labial palps 2-segmented), i.e. *brachypterus* (Mercet) and *deluchii* Viggiani, both from Europe. These differ significantly from *macadamiae* in having the mid and hind tibiae each with a pair of distinct dark brown rings, the head about 4X as wide as the frontovertex and the scape about 3.X as long as broad. *Metaphycus brachypterus* also has the mouth margin and gena brown and *deluchii* has linear sensilla on F5.

Of the remaining species of *Metaphycus* belonging to the *alberti* group, the most similar in general appearance and habitus is *helvolus* Compere, females of both species being generally yellow in appearance with the scape broadened and flattened and mostly dark brown, apex of clava pale yellow, occiput and pronotum marked dark brown, mid tibia with a faint brown subbasal ring, funicle with linear sensilla only on F6, and male with pores scattered along inner margin of torulus. The female of *macadamiae* differs from *helvolus* in having a brown streak on the gena, and the scape about 3X as long as broad, whereas in *helvolus* the gena is completely pale yellow and the scape is 2.5X as long as broad. The male of *macadamiae* differs from that of *helvolus* in having fewer than five pores along the inner margin of the torulus, and the funicle segments are nearly 2X as broad as long, whereas in *helvolus* there are at least 10 pores and the funicle segments are subquadrate.

In the key to the Hawaiian *Metaphycus* species [33], *macadamiae* runs to couplet 6 which includes "sp. near *claviger*" and *alberti* (Howard). It runs best to "sp. near *claviger*" because the scape is said to be about 3X as long as broad whereas in *alberti* the scape is said to be about 4X as long as broad (actually about 2.5X as long as broad in *claviger* and 3X as long as broad in *alberti*). As both *alberti* and *claviger* are very similar to *macadamiae* and probably originate from Australia, *macadamiae* is compared to both below.

Females of *macadamiae* differs from those of *alberti* and *claviger* in being smaller, generally less than 0.8mm long (mostly at least 1mm long in *alberti* and *claviger*), having a pale brown mark on gena (absent in *alberti* and *claviger*), linear sensilla only on F6 (F5 and F6 in *alberti*); head, side and venter of thorax white (orange in *alberti* and *claviger*), mid and hind tibiae each with a pale brown subbasal ring (legs immaculate in *alberti* or *claviger*), head usually about 3X as wide as the frontovertex (rarely as much as 3.6X, but at least about 3.8X *alberti* or *claviger*) and ovipositor slightly longer than mid tibia (about 0.8–0.9X as long in *alberti* and *claviger*). Males differs from those of *alberti* and *claviger* in having the scape virtually uniformly white (pale orange with a distinct pale brown median band in *alberti* and with dorsal and ventral margins brown in *claviger*), from *claviger* in having the gena pale pink (brown in *claviger*), from *alberti* in having at most only 4 pores along inner margin of torulus that do not extend past upper margin (at least 8 in *alberti* some of which extend past upper margin) and from *claviger* in having F6 strongly transverse, only about 0.6X as long as broad and only slightly larger than F5 (in *claviger* subquadrate, nearly as long as broad and much larger than F5).

#### Molecular analysis

The paucity of DNA sequences for *Metaphycus* species in GenBank or elsewhere, coupled with the relative shortness of our sequences have precluded the need for any phylogenetic or even phenetic analyses. A Genbank BLAST of our 444 bp 28S ribosomal sequence suggests some proximity to *M*. *helvolus* (assuming correct identification), which is also suggested by morphology (see above). The top 8 similar sequences are all *Metaphycus* species. *M*. *helvolus* in Genbank has 97% query cover with 94% identical bases, suggesting quite some genetic distance. Our 555 bp COI contig of 6 sequences BLASTs to “Encyrtidae sp.” with 91% query cover and 92% identity. It would appear that *M macadamiae* is not closely related to any species with sequences currently deposited in Genbank.

#### Comparison of *Metaphycus macadamiae* with *M*. *dispar* (Mercet)

To the untrained eye, as was revealed during the review process of this paper, some superficial similarity between *M*. *macadamiae* and *M*. *dispar* could be considered. The main and obvious differences in their appearance are listed below. Also worthy of consideration are the facts that *M*. *dispar* is known only from Coccidae, is a Palaearctic species (introduced into Califormia), and does not occur in Australia.

*M*. *dispar* differs notably from *M*. *macadamiae* in having linear sensilla on F5; the gena and mouth margin lack brown marks; the notauli reach almost reach half way down the mesoscutum. It is also distinctly paler and in general larger.

Other differences are as follows (the condition in *M*. *macadamiae* is given first, with *M*. *dispar* following in parentheses in red typeface):

Female: Head mostly white (yellow/pale orange) with occiput above foramen dark brown and gena with a slightly elongate pale brown mark between base of mandible and eye (yellow/pale orange); mouth margin with a slender brown mark above base of mandible and another below torulus (yellow/pale orange); antenna with radicle white (brown); scape white with a broad median band about half its length on both inner and outer surfaces, connected ventrally but narrowly separated dorsally (continuous); mesoscutum pale orange (dark orange); metanotum dusky pale orange; tegula translucent white, apex pale grey; side and venter of thorax white (pale orange); mid and hind tibiae each with an extremely faint pale brown subbasal ring (only mid tibia with pale ring).

Male: Antennal torulus with from 1 to 4, widely spaced, associated pores along inner margin (cluster of c9 pores ventro-laterally); antenna with only F6 with linear sensilla (F5+F6).

## Discussion

Host specificity tests and biological studies in Hawaii will be published elsewhere when nomenclature of this parasitoid is officially published. We anticipate that *M*. *macadamiae* will be a useful agent in the biocontrol programmes against MFC in Hawaii and South Africa. In April 2017 severe infestations of MFC were observed in the Barberton valley in Mpumalanga, South Africa. The impact of this new pest on the local macadamia industry may take some years to reach the infestation level in Hawaii. However, it is an important quarantine organism and researchers are advocating care to prevent the movement of infested plant material to reduce the risk of spreading the pest amongst orchards. Although it was initially thought the infestation was contained in Barberton where the pest was first found, it spread within a month to White River plantations about 63 Km north of Barberton, presumably through infested plant material.

Spread in Hawaii is relatively slow, and the scale tends to stay in the same tree. But observations in White River contradict this as there was considerable spread to adjoining trees. South African Entomologists are waiting for the release of *M*. *macadamiae* in Hawaii to get a starter colony for their studies (https://macadamiasa.co.za/2019/02/19/beware-the-felted-coccid/.

## Supporting information

S1 Video (AVI)

S2 Video (AVI)
